# High‐Efficiency, Matrix Interference‐Free, General Applicable Probes for Bile Acids Extraction and Detection

**DOI:** 10.1002/advs.201800774

**Published:** 2018-10-21

**Authors:** Shuyao Huang, Jiating Zheng, Qian Yang, Guosheng Chen, Jianqiao Xu, Yong Shen, Yimin Zhang, Gangfeng Ouyang

**Affiliations:** ^1^ MOE Key Laboratory of Bioinorganic and Synthetic Chemistry School of Chemistry Sun Yat‐Sen University Guangzhou Guangdong 510275 China; ^2^ Urology Department Sun Yat‐Sen University Sixth Affiliated Hospital Guangzhou Guangdong 510000 China

**Keywords:** β‐cyclodextrin, bile acid, host–guest interactions, matrix interference‐free, solid phase microextraction

## Abstract

Although bile acids (BAs) have been suggested as important biomarkers for endocrine diseases, the identification and quantification of different BAs are still challenges due to their enormous species and wide range concentrations. Herein, a copolymer probe based on β‐cyclodextrin (β‐CD) is fabricated through a simple in‐mold photopolymerization for the selective extraction of BAs. Through the unique stereochemical affinity between BAs and the cavity of β‐CD, the custom probe shows superior enriching capacities to series BAs. Moreover, the outstanding extraction ability is proved to be consistent in various interfering conditions, including pH changing and the addition of complex matrix. Further comparison shows that the stereostructure of the nucleus of BAs plays a vital role during the formation of the β‐CD/BA complex, indicating the potential for efficient extraction of other BAs, including their structural analogues or some unknown ones. The developed probe is used for solid phase microextraction, and the limits of detection are lower than 0.075 ng mL^−1^ by coupling to high performance liquid chromatography‐tandem mass analysis. The results in this study highlight the potential for effective improvement of immediate detection and profiling of BAs in real samples, which will make a tremendous impact in the analytical field or clinical diagnosis.

## 1 Introduction

Bile acids (BAs) are the amphipathic products of cholesterol metabolism in vertebrates,^1^ playing fundamental roles in physiological processes,^2^, ^3^ or acting as signaling molecules of systemic endocrine functions.^3^, ^4^ It has been suggested for long that changes of BAs levels could be linked to tumor formation^5^, ^6^ in different organs, such as the esophagus,^7^ stomach,^8^ and small intestine^9^ or liver injury.^10^ Herein, comprehensive profiling and quantification of BAs will be vital for the diagnostic, therapeutic, and prognostic management of the related diseases.

As a matter of fact, efficient approaches aiming at differentiating and detecting the spectrum of BAs were presented,^11^, ^12^, ^13^, ^14^ where mass spectrum (MS),^11^ tandem mass spectrum (MS/MS),^15^ or other high resolution mass spectrum^16^ were the dominated analysis tools. However, some of these multifarious BAs biomarkers are at extremely low levels (less than 0.1 × 10^−9^ m)^2^ while the coexisting distracting molecules present at much higher abundance, thus their signal might be undetectable or covered. Meanwhile, the high content of inorganic salts in living system will further suppress the signal readout.^17^ Conceivably, engineering a sensitive analytical platform integrated high throughput screening and matrix interference‐free merits will advance the ideas to profile and detect the BAs. Nevertheless, no efforts have been devoted to addressing this topic. The major challenges can be attributed to the following two aspects: 1) Series of reactions^12^ including dehydration, dehydrogenation, dehydroxylation, loss of carbon monoxide or carbon dioxide and epimerization occurred on BAs in the living system, and diversified the BAs with different physicochemical properties. As a result, the common polarities‐based separation approaches, such as liquid extraction‐ and solid phase extraction‐assisted methods,^18^, ^19^ suffered from poor selectivity and coverage toward BAs analysis. 2) The selectivity‐improved approach, such as molecularly imprinted strategy was limited by the single‐component analysis, so that the high throughput screening was a continuous challenge. Meanwhile, the template leakage problems greatly hinder the practical application especially in low level target detection.^20^, ^21^

Recently, β‐cyclodextrin (β‐CD) was reported to act as a host to form stable host–guest inclusion complex with BAs, since the size and shape of BAs fit well with the cavity of β‐CD through van der waals interaction or hydrogen‐bond interaction.^22^, ^23^, ^24^, ^25^, ^26^, ^27^ We also noted that these numerous BAs exhibited similar molecular structures, comprising a cyclopentanoperhydrophenanthrene nucleus and a carboxylate side‐chain. It is reasonable to consider the new stereochemistry‐induced strategy as an optimal choice for an efficient and broad‐spectrum extraction of BAs from complex matrix.

Herein, we presented a β‐CD based stereochemistry‐induced solid phase microextraction (SPME) probe for BAs determination, featuring the merits of high throughput screening, matrix interference‐free and excellent selectivity. SPME has been considered a promising method to promote the sample preparation efficiency especially when treating highly complex samples, because it integrates separation, enrichment, and purification into one step.^28^ The elaborate β‐CD units was perfectly fixed within the SPME coating via a facile in situ photopolymerization strategy, which could significantly improve the accessibility of the recognition sites and enhance the matrix interfering‐resistance capacity simultaneously. Here polyethylene glycol 400 diacrylate (PEG400DA) was adopted as the cross‐linker and the introduction of polyethylene glycol (PEG) section endowed the copolymer good hydrophilicity as well as good biocompatibility. We demonstrated the proposed SPME probe was capable to selectively and efficiently extract BAs even under pH changing or the interfering substances coexisting situations. Importantly, extensive BAs with diverse physicochemical property were enabled to be extracted owing to the stereochemistry‐induced extraction principle.

## 2 Results and Discussions

### 2.1 Fabrication, Optimization, and Characterization of the PEG/β‐CD Probe

**Figure 1:**
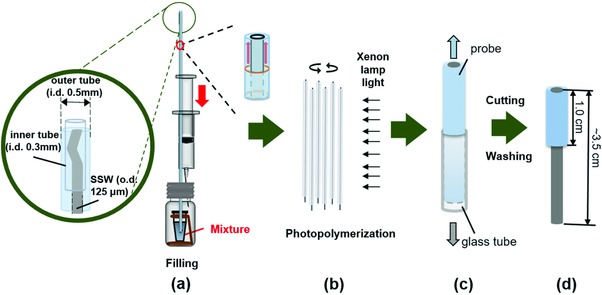
The schematic diagram of fabrication procedure of copolymer probe.

The schematic diagram of fabrication procedure of the copolymer probe was presented in **Figure** **1** and details could be found in the Experimental Section. Vinyl group was first derived on β‐CD through a simple esterification reaction (Figure S1, Supporting Information) and the monomer for copolymerization, β‐cyclodextrin methacrylate (β‐CD‐MA), was successfully obtained. According to the ^1^H NMR spectroscopy (Figure S2, Supporting Information), majority of the spectroscopy of the derivatives was consistent with β‐CD, however, the appearance of peaks at 5.97 ppm (s, –OOCC(CH_3_)C***H*** *_2_*–), 5.60 ppm (s, –OOCC(CH_3_)C***H*** *_2_*–), and 1.84 ppm (s, –OOCC(C***H*** *_3_*)CH_2_–) indicated the successful introduction of vinyl group. The derivatization took place on 6‐OH rather than 2‐OH, or 3‐OH, which led to a slight declination at 4.44 ppm (t, –R_1_R_2_CHC***H*** *_2_*OOC–, 6‐OH), as well as slight chemical shift increase on 6‐H (details could be found in the Supporting Information), probably due to the 6‐OH exhibited lower steric hindrance and intramolecular hydrogen bonds were formed between 2‐OH and 3‐OH.^29^ Polyethylene glycol 400 diacrylate (PEG400DA) was then used as the cross‐linker to endow the copolymer higher water compatibility, because PEG has been considered as an excellent material for medical disciplines due to its high hydrophilic and biocompatibility.^30^ After polymerization, the probe was immersed in water and then methanol to remove the porogen and residual monomers.

**Figure 2:**
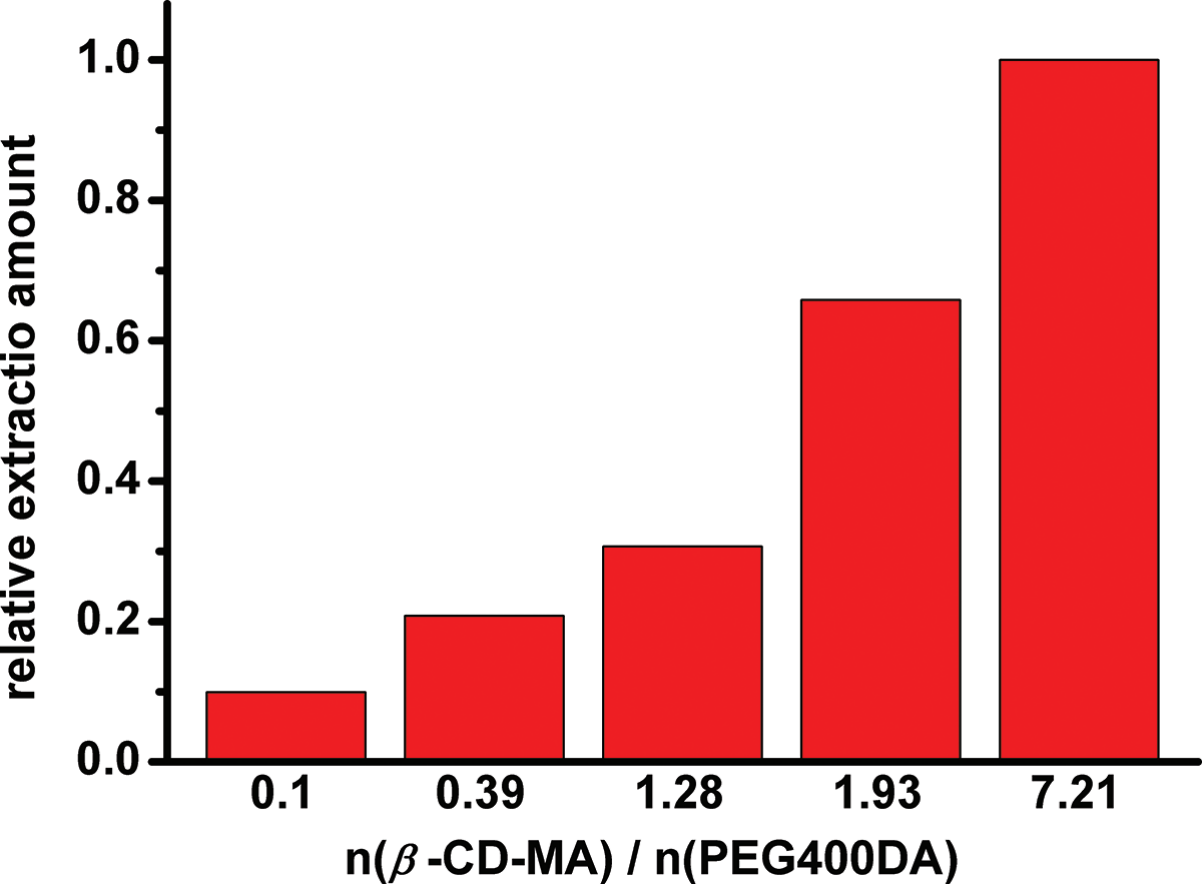
Optimization results by changing the ratio of two monomers for polymerization. Statistics were calibrated by the highest extraction amounts.

In order to get a better performance for BAs extraction, the composition of copolymer was optimized by adjusting the ratio of two monomers, β‐CD‐MA and PEG400AD. Ursodeoxycholic acid (UDCA), a typical dihydroxycholanic acid, was used as the model analyte for the evaluation. According to **Figure** **2**, the extraction amount of UDCA was increased along with the proportion of β‐CD‐MA. However, the copolymer became soft and semisolid when continually raising the ratio of β‐CD‐MA, probably because the lack of crosslinkers resulted in the decrease of polymerization degree. The final ratio of β‐CD‐MA and PEG400AD was set as 7.21 in the following experiments.

**Figure 3:**
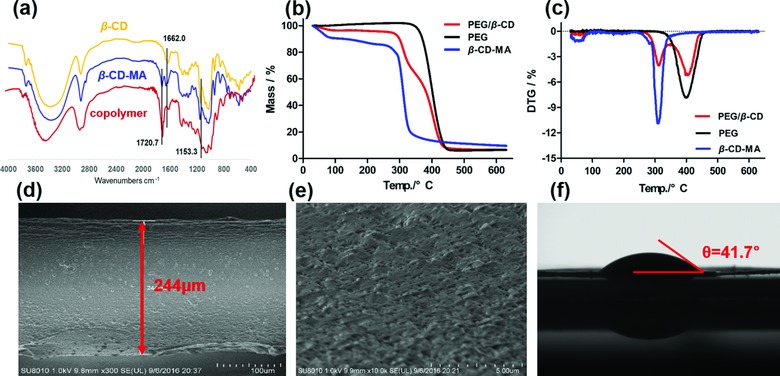
a) FT‐IR spectra of ingredient β‐CD, the monomer β‐CD‐MA, and the β‐CD/PEG copolymer. b) TG graphs and c) DTG graphs of as‐prepared monomer (β‐CD‐MA), PEG, and synthesized copolymer (β‐CD/PEG). d,e) SEM images of the whole probe and its surface. f) Contact angel of water on the thin film copolymer.

The characterization results could be found in **Figure** **3**. According to the FT‐IR spectrum (Figure 3a), the adsorption bands at 1655 and 1720 cm^−1^ were associated with the stretching vibration of C=C bond and C=O bond respectively, while no additional bands above 1750 cm^−1^ (referring to the stretching vibration of C=O bond in the reactant anhydride) was observed, demonstrated that the monomer β‐CD‐MA was successfully synthesized. The disappearing of adsorption band around 1655 cm^−1^ and the slight blue shift at 1720 cm^−1^ indicated the polymerization was successful and the residual unreacted monomers were completely removed. We subsequently compared the thermogravimetric (TG, Figure 3b) and the derivative thermogravimetric (DTG, Figure 3c) curves of the copolymer material and two monomers. The mass loss of PEG/β‐CD copolymer mainly occurred at regions of 250–345 and 345–460 °C, at which β‐CD‐MA and PEG decomposed respectively. The mass loss proportions in different regions revealed that the ratio of β‐CD consisted in the copolymer was about 57%, which was much less than its ratio in the reaction system. High viscosity of the reactant mixture and the steric hindrance brought by β‐CD might hinder the effective interaction between monomers. To ensure the ratio of β‐CD in the ultimate probe, thoroughly mixing of the reactants before curing was crucial.

In order to get SPME probes with uniform copolymer coating, assembled molds (Figure 1a) were used for the in suit polymerization according to previous works with slight modification.^31^, ^32^ The scanning electron microscope (SEM) image (Figure 3d) of the custom probe indicated that uniform surface was obtained, and the thickness of the coating was about 59 µm by calculation (the outer diameters of the probe and the inner stainless steel wire were 244 and 125 µm, respectively). Irregular holes on the surface of the probe were observed due to the addition of porogen during polymerization (Figure 3e). Benefited from the porous structure as well as the existence of PEG, the material showed good affinity to water, as the contact angle of water against the copolymer was about 41.7° (Figure 3f).

### 2.2 High‐Efficient Extraction to UCDA

**Figure 4:**
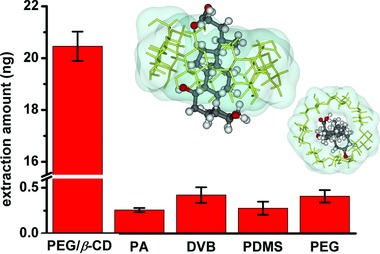
Extraction capacity comparison between the copolymer probe to commercial SPME fibers (DVB and PA) and two custom fiber (PDDA dispersed PDMS fiber and the pure PEG fiber) (*n* = 6). Insets are the front view and top view of the β‐CD/UDCA complex obtained by molecular docking.

Experiments were operated in phosphate buffer solution to evaluate the extraction efficiency of the probe by coupling direct immersion SPME with high performance liquid chromatography‐tandem mass (HPLC‐MS/MS) analysis (see details in the Experimental Section). As a novel method integrates separation, enrichment and purification into one step, SPME has been successfully employed for sample preparation in many bioanalysis studies.^28^ The enrichment factor of the probe to UDCA was evaluated to be about 2.98 × 10^7^, indicating its outstanding extraction capacity. Subsequently, the extraction amount of the PEG/β‐CD copolymer probe to UDCA was compared with commercial or other two custom SPME fibers (**Figure** **4**). Commercial divinylbenzene (DVB) and polyamide (PA) fibers were selected due to their high affinities to polar compounds. Besides, a poly diallyldimethylammonium chloride microcapsules dispersed polydimethylsiloxane probe (PDMS@PDDA) was also used for extracting UDCA based on the electrostatic interaction between the target analytes and the cationic polyelectrolyte. The PDMS@PDDA probe was fabricated according to a previous work^31^ with a slight modification. Nevertheless, the copolymer probe showed superior extraction capacity to UDCA comparing to the other probes (80 times to PA, 49 times to DVB, and 74 times to PDMS@PDDA).

Moreover, pure PEG probe was fabricated through the in‐mold polymerization strategy, without adding β‐CD‐MA in the polymerization system. Still, poor extraction capacity was observed for the PEG fiber, which was strong evidence demonstrated β‐CD made the major contribution for the superior extraction capacity of the probe to UDCA. A simple docking study was operated on UDCA and β‐CD by using Discover in Materials Studio (Accelrys, Inc., San Diego, CA, USA)).^33^ The docking result (see in the front view and top view of the complex in Figure 4) indicated that the UDCA molecule perfectly fitted in the cavity of β‐CD, which further confirmed the host–guest interaction played the vital role in the extraction.

### 2.3 Matrix Interfering Resistance of the Probe

Direct extraction without pretreatments (for example, pH adjustment, protein precipitation, etc.) to biosamples is preferred as it guarantees the detection reflecting actual levels of target analytes in samples. Herein, the resistance of the custom probe to matrix interferences was evaluated.

It is known that the ionization of BAs, including the free or conjugated ones and their derivatives, will be greatly influenced by the pH condition. According to Equation (1)

(1) $$\log\frac{\left[\mathrm{HA}\right]}{\left[A^{-}\right]}=\;pKa-\mathrm{pH}$$

**Figure 5:**
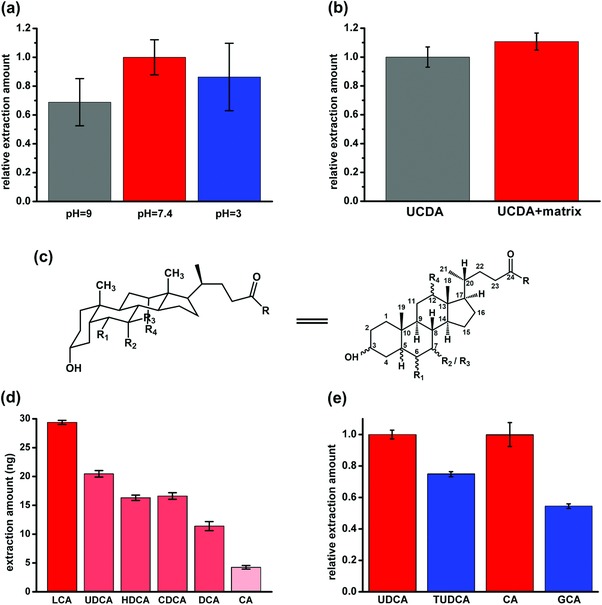
Extraction amount of the copolymer probe to UDCA under a) pH interfering (statistics were calibrated by the value obtained in solution with pH = 7.4, *n* = 6), and b) matrix effect (statistics were calibrated by the values obtained in matrix‐free solution, *n* = 6). c) The general molecular structure of bile acids. d) Extraction amounts of BAs with different nuclear structure on the probe (*n* = 6). e) Comparison of the affinity of free (UDCA, CA) and conjugated BAs (TUDCA, GCA) to the copolymer probe (statistics were calibrated by the value of the corresponding free BAs, *n* = 6).

The theoretical ratio between unionized and ionized analyte differs by orders of magnitude when the pH of solution changes greatly. For traditional methods based on polarity, the majority of extracted UDCA is in the unionized state, which was also proved by the marked increase of log*D* value as the pH declines (**Table** **1**). As a result, pH adjustment is necessary during traditional sample preparation procedures in order to obtain higher extraction amounts. In this study, the extraction capabilities of the copolymer probe to UDCA in different solution, of which the pH value varied from 3 to 9, were investigated. Only slight vibration of the extraction amount was observed when changing the solution to acid or alkaline conditions (**Figure** **5**a). Owing to the unique stereochemical selectivity, not only the unionized state target compounds but also the ionized ones were extracted on the newly developed copolymer probe. As a result, the probe showed high potential for a successful direct extraction from untreated biosamples, which will improve the detection efficiency by reducing the sample pretreatment time.

**Table 1:** The structures and physical properties of eight bile acids investigated in this work

	Stereochemical structurea)					log*D* [25 °C]b)			Molecular weight	p*K*a [25 °C]b)
	R_1(α)_	R_2(α)_	R_3(β)_	R_4(α)_	R	pH = 3	pH = 7.4	pH = 9		
CA	H	OH	H	OH	OH	2.44	−0.38	−0.81	408.57	4.76 ± 0.10
GCA	H	OH	H	OH	NHCH_2_COOH	1.31	−1.77	−1.91	465.62	3.58 ± 0.10
HDCA	OH	H	H	H	OH	3.66	1.06	0.43	392.57	4.76 ± 0.10
CDCA	H	OH	H	H	OH	3.66	0.92	0.41	392.57	4.76 ± 0.10
UDCA	H	H	OH	H	OH	3.66	1.06	0.43	392.57	4.76 ± 0.10
TUDCA	H	H	OH	H	NHC_2_H_4_SO_2_OH	−0.47	−0.47	−0.47	499.70	1.42 ± 0.50
DCA	H	H	H	OH	OH	2.44	−0.38	−0.81	392.57	4.76 ± 0.10
LCA	H	H	H	H	OH	4.95	2.35	1.71	376.57	4.76 ± 0.10

^a)^ Detail positions could be found in Figure 5c

^b)^ Statistics were obtained from stimulation results by MarvinSketch (ChemAxon).

Another obstacle for the analysis of biosamples is the extremely complex matrix. Various endogenous substances in biofluids might cause competitive adsorption with target analytes, thus greatly hinder the extraction. To value the matrix effect to the extraction, a mixing solution containing 200 ng mL^−1^ UDCA and about 21 times total concentration of interfering substances (including proteins, organic acids, amino acids, and carbohydrates etc.) was used for extraction. The extraction amount on the probe in the mixing solution showed good consistent to the value obtained in pure UDCA solution (Figure 5b). In other words, the addition of matrix compounds merely interfered in the extraction of target analyte on the copolymer probe.

Protein fouling is another difficulty should be overcome for direct extraction in biofluids, because the fouling protein might block the binding sites and lead to performance reduction. In this study, PEG, a well‐known biocompatible material, was used as the crossing linker in polymerization to avoid protein fouling, which was subsequently proved to be successful in the following real sample detection, as no obvious peak was found when the *m*/*z* value was over 1000 (Figures S3 and S4, Supporting Information).

### 2.4 General Applicability of the Probe to Extensive BAs

The hydroxylation occurred at C3, C6, C7, and C12 sites as well as the conjugation of glycine or taurine at the C24‐carboxyl group (Figure 5c) are two main factors lead to the structural diversities of human BAs.^12^ In the present work, seven other BAs with different structure and conjugated groups, of which the detail structures were presented in Table 1, were selected for further evaluation.

**Figure 6:**
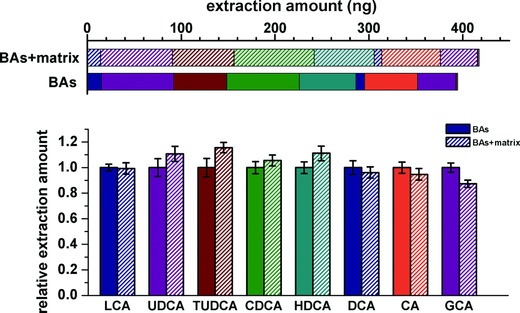
Evaluation of the matrix effect on the total (upper bar charts) and individual extraction amounts of eight studied BAs on the probe (*n* = 6), statistics in the bottom histogram were calibrated by the values obtained in the matrix free solution respectively.

The extraction capacity of the probe showed declining tendency as the hydroxyl groups gradually increased on the tetracyclic nucleus, while four geometric isomers, UDCA, deoxycholic acid (DCA), hyodeoxycholic acid (HDCA), and chenodeoxycholic acid (CDCA), preformed similar affinity to the probe (Figure 5d). On one hand, more hydroxyl groups on the nucleus meant higher steric hindrance when the molecule fit into β‐CD, on the other hand, the hydrophobic cavity of β‐CD would showed lower affinity to analytes of which the nucleus were more hydrophilic, for instant, the cholic acid (CA). Interestingly, only slight decline was found for conjugated BAs, for example tauroursodeoxycholic acid (TUDCA) and glycocholic acid (GCA), when comparing with the unconjugated ones (UDCA and CA) owning the same nucleus respectively (Figure 5e). Moreover, the good affinity of the probe to all studied BAs showed considerable resistance to the matrix interfering (**Figure** **6**), as the extraction amounts of every BAs or their total mount on the probe (the upper bar chart) kept consistence after adding the matrix, respectively.

From the aforementioned results, we could draw a conclusion that the stereostructure of the nucleus, rather than the conjugation at C24‐carboxyl group plays the vital role during the formation of β‐CD/BA complex. Although dehydrogenation of hydroxyl groups also generates certain species of BAs, the keto groups on these products process similar effect to the hydroxyl groups, therefore will be extracted by the β‐CD through the same mechanism. Considering there are limit species of BAs contain more hydroxyl groups on the nucleus than CA and the superior performance revealed by the mentioned results (Figure 5d,e), the copolymer SPME probe exhibits great potential in efficiently extracting most species of BAs. Therefore, the work presented a promising way for high throughput screening, as the simple SPME procedure could be conducted by a robotic autosampler that enabled simultaneous preparation,^34^ which greatly shorten sample pretreatment time and less manpower was required. As a result, great improvement to the identification or selective determination of different BAs in biofluids will be brought, which is believed to be of great importance for disease diagnosis, biomarker profiling, or metabolomics studies.

### 2.5 Real Sample Determination

Finally, the SPME probe was coupled with HPLC‐MS/MS analysis, and good analytical performances were obtained. Outstanding enrichment factors of the probe to all studied BAs led to the high extraction capacity, so that the limits of detection (LODs) of the method for eight BAs were all below 0.075 ng mL^−1^, which is much lower than the reported works (Table S1, Supporting Information). Besides, the wide linear ranges, good reproducibilities (for intrafiber and interfiber) ensured the feasibility of the developed method (**Table** **2**; Figure S5, Supporting Information).

**Table 2:** The method evaluation results of BAs determination using the copolymer SPME probe coupling to HPLC‐MS/MS

		CA	LCA	TUDCA	UDCA	CDCA	GCA	HDCA	DCA
Enrichment factor [10^7^]		0.621	4.28	2.23	2.98	2.42	0.39	2.38	1.66
Sampling rate [10^−3^ ng min^−1^]		6.40	24.83	17.53	26.81	23.60	3.54	23.11	16.42
LOD [ng mL^−1^]		0.028	0.015	0.003	0.036	0.075	0.056	0.026	0.028
Linear range [ng mL^−1^]		0.1–500	0.05–500	0.01–500	0.12–500	0.25–500	0.20–500	0.09–500	0.09–500
*R* ^2^		0.9983	0.9806	0.9894	0.9994	0.9989	0.9998	0.9882	0.9971
Reproducibility [%]	Intrafiber	4.6	4.4	4.1	5.9	4.2	2.8	5.7	4.3
	Interfiber	12.5	13.3	8.9	8.9	10.1	7.5	7.6	10.2

Real sample detection was operated on human urine sample by using the present method, and six of target BAs were successfully detected (Table S2, Supporting Information). Due to the outstanding matrix‐resistance of the custom probe, the extraction was directly operated in urine samples without further pretreatment. TOF‐MS analysis of the eluent showed that larger molecules were successfully removed, as no obvious peaks were found when the *m*/*z* was over 1000, while peak around 2900 was observable when using the traditional methanol precipitation method (Figure S3, Supporting Information). The recoveries of eight BAs are around 80.74–108.47%, which also indicated the reliability of the method.

## 3 Conclusion

A novel PEG/β‐CD copolymer SPME probe was fabricated through a simple in‐mold photopolymerization method and utilized for extracting BAs in biofluids. Different from traditional separation methods based on polarity similarity, the developed probe showed extremely high extraction capacity to BAs because of the unique stereochemical selectivity between BAs and the cavity of β‐CD. Moreover, the high enrichment efficiency was proved to be free from the matrix interfering, including the pH changing, the competitive extraction by other endogenous molecules and the protein fouling, which is vital for direct extraction from the highly complex biofluid samples. Further comparison demonstrated that the stereostructure of the nucleus of BAs played the vital role during the formation of β‐CD/BA complex, indicating the potential for efficient extraction of unknown BAs or their structural analogues. It is believed to bring significant improvement to BAs identification by the developed method, which is of great importance in disease diagnosis, biomarker profiling, or metabolomics studies. As in vivo SPME has been well‐developed and widely applied in analyzing pharmaceuticals or endogenous substances,^35^, ^36^ in vivo detection or instant determination based on this method is expected to be realized in the future.

## 4 Experimental Section

*The Synthesis of Beta‐Cyclodextrin Methacrylate (β‐CD‐MA)*: Vinyl group was derived on β‐CD by a simple esterification reaction (Figure S1, Supporting Information). Β‐CD (5.67 g, 0.005 mol) was weighted and dissolved in dry dimethyl formamide (DMF, 30 mL) in a flask. Then methacrylic anhydride (7.70 g, 0.050 mol) was added and the mixture was stirred at 500 rpm for 12 h at 80 °C under reflux. After the reaction finished, residue was filtered and large amount of chloroform was poured into the filtrate. The precipitation was three times washed by acetone, then collected and dried in vacuum at 20 °C for 24 h. The product was stored at −20 °C to avoid oxidation until use.

*The Preparation of β‐CD/PEG Copolymer Probe*: β‐CD‐MA (0.12 g) was dissolved in DMF (0.06 g) in a 0.5 mL centrifuge tube, then 1‐hydroxycyclohexyl phenyl ketone solution (3% in DMF, 0.03 g) was added and stirred roughly. A mixture of polyethylene glycol 400 diacrylate (PEG400DA) and polyethylene glycol 200 (PEG200) was prepared (PEG400AD:PEG200 = 1:2, w/w). Then the mixture (0.06 g) was added in the centrifuge tube and stirred evenly in dark. The preparation procedure (Figure 1) of the custom SPME fiber was based on a previous study with slight modification.^32^ SSWs were cut into sections about 3.5 cm and ultrasonic washed in acetone. Then the dried SSW was inserted into capillary tube (i.d. 0.5 mm) which acted as the mode for fiber preparation. To keep the SSW and the outer capillary tube concentric, thinner capillary tube (i.d. 0.3 mm, length 1 mm) was inserted between the outer tube and the SSW at both two ends. The centrifuge tube containing the mixture was set in a 10 mL sample vial and the assembled capillary was pierced through the sealing gasket until one end was immersed in the mixture. Then the mixture was pressurized into the tube by pumping air into the vial using a hypodermic syringe (Figure 1a). The mixture‐filled capillary tube was immediately placed under a xenon lamp (CEL‐HXUV300, Beijing China Education Au‐light Co., Ltd) for 90 s (Figure 1b) so that the β‐CD/PEG coating was in suit symmetrically grown on the SSW. The probe can be easily pushed out from the outer tube (Figure 1c) and then immersed in a large amount of water and methanol, with a gentle shake for 1 h and 30 min respectively, to remove the unreactive substances and the residual monomers. Subsequently, the coating was scratched with a knife with 1.0 cm copolymer remaining at one end (Figure 1d). Finally the obtained probes were dried in the air and kept under seal until used. Custom PEG fibers were prepared in the same way without adding β‐CD‐MA in the polymerization for comparison.

*Material Characterization*: ^1^H NMR analysis was conducted on a Bruker Avance III superconducting Fourier transform nuclear magnetic resonance spectrometry by using the DMSO‐*d6* as the solvent. Fourier transform infrared (FTIR) spectra were recorded on a Bruker EQUINOX 55 Fourier transform infrared spectrometer. Thermogravimetric analysis was conducted from 30 to 850 °C with the heating rate of 5 °C min^−1^ under N_2_ protection on thermogravimetric analyzer (TG 209 F3 Tarsus) from NETZSCH‐Gerätebau GmbH. The contact angle of water on the copolymer material was detected by an optical contact angle analysis system (OCA200) DataPhysics Instruments GmbH. SEM images were obtained from a SU8010 ultrahigh resolution field emission scanning SEM (Hitachi, Ltd., Japan).

*Method Development and Evaluation*: Extractions were conducted by immersing the probe in 8 mL prepared solutions for 30 min under vibration (600 rpm). After the extraction finished, the probes were washed by deionized water for 5 s and gently wiped, then immersed in 80 µL of methanol for desorption (60 min) on vibration (400 rpm). All the desorption solution was stored under −20 °C until instrumental analysis. An Agilent 1260 HPLC system (Agilent Technologies, CA, USA) coupled to an Triple Quad 4500 triple‐quadrupole tandem mass spectrometer with an ESI source (Applied Biosystems/MDS Sciex, MA, USA) was operated in the negative ion mode for analyzation. Detail instrumental states could be found in Tables S3 and S4 in the Supporting Information. In order to evaluate the linear ranges and LODs of the method, solutions with a wide range concentrations of BAs (0.05, 0.1, 1, 10, 20, 50, 100, 500 ng mL^−1^ in phosphate buffer solution (PBS)) were used in extraction. Subsequently, the interfiber and intrafiber reproducibilities of the probe were also determined by sampling in 20 ng mL^−1^ BAs solution (*n* = 6). Sampling‐rate calibration method^37^ was used in this study for methodology validation according to Equation (2)

(2) $$R_{s}=\frac{n}{C_{s}\cdot t}$$

where *R*_s_ referred to the sampling rate, *n* was the extraction amount of target BAs in the fiber, *C*_s_ referred to the BAs concentrations in sample matrices and *t* was the extraction time. After getting the sampling rates of eight BAs in PBS solution based on the standard lines (Figure S5, Supporting Information), verification experiments were conducted by extracting BAs from solution (20 ng mL^−1^) for 10 and 50 min respectively. The similar extraction amounts obtained by the actual experiment and calculated according to Equation (2) confirmed the method to be reliable (Figure S6, Supporting Information).

*Real Sample Detection*: The custom copolymer probes were directly immersed in 2 mL human urine mixing samples (*n* = 6) for extraction, and the subsequent extraction and desorption procedures were the same as those mentioned above.

## Conflict of Interest

The authors declare no conflict of interest.

## Supporting information

Supplementary
