# Supplementary material for: High‐Efficiency, Matrix Interference‐Free, General Applicable Probes for Bile Acids Extraction and Detection
Source: Adv Sci (Weinh). 2018 Oct 21;5(12):1800774. doi: 10.1002/advs.201800774 (PMC6299822; doi:10.1002/advs.201800774)
Supplement: Supplementary file 1 — Supplementary [file ADVS-5-1800774-s001.pdf]

Copyright WILEY-VCH Verlag GmbH & Co. KGaA, 69469 Weinheim, Germany, 2018.

Supporting Information

for *Adv. Sci.,* DOI: 10.1002/advs.201800774

High-Efficiency, Matrix Interference-Free, General Applicable Probes for Bile Acids Extraction and Detection

Shuayo Huang, Jiating Zheng, Qian Yang, Guosheng Chen, Jianqiao Xu, Yong Shen, Yimin Zhang, and Gangfeng Ouyang**

Copyright WILEY-VCH Verlag GmbH & Co. KGaA, 69469 Weinheim, Germany, 2018.

Supporting Information

High-efficient, matrix interference-free, general applicable probes for bile acids extraction and detection

Shuayo Huang, J3iating Zheng, Qian Yang, Guosheng Chen, Jianqiao Xu, Yong Shen, Yimin Zhang, Gangfeng Ouyang**

1. Reagents and materials

Polyethylene glycol 400 diacrylate (PEG400DA, containing 400-600ppm MEHQ), methacrylic anhydride (MA, 94%, containing 0.2% topanol), cholic acid (CA, 98%), lithocholic acid (LCA, 95%), deoxycholic acid (DCA, 98%), glycocholic acid (GCA, 97%), chenodeoxycholic acid (CDCA, 98%), hyodeoxycholic acid (HDCA, 98%), tauroursodeoxycholic acid (TUDCA, 98%), ursodeoxycholic acid (UDCA, 99%), palmitic acid (97%), lauric acid (99.5%), peroxidase, methanol and dimethylformamide (DMF) were all obtained from Aladdin Reagent Company (Shanghai, China). Polyethylene glycol 200 (PEG200) was purchased from Alfa Aesar (UK). 1-hydroxy cyclohexyl phenyl ketone and the amino acid standard (L-alanine: 1.25 μmol/mL in 0.1 N ammonium chloride solution; L-arginine, L-aspartate, l-cystine, L-glutamic acid, glycine, L-histidine, L-isoleucine, L leucine, L-lysine, L-methionine, L-phenylalanine, L-proline, L-serine, L-threonine, L-tyrosine, L-valine: 2.5 μmol/mL in 0.1 N ammonium chloride solution) were purchased from Sigma-Aldrich Co. Ltd. (St. Louis, USA). Beta-cyclodextrin (*β*-CD, 99%), myristic acid (99%) and Dexamethasone (96%) were obtained from J&K Scientific (Beijing, China) respectively. Stainless steel wires (SSWs, 125 μm in diameter) were purchased from Small Parts Inc.

(Miami Lakes, USA). Human urine was obtained from Centre for Disease Prevention and Control of Guangdong Province (Guangzhou, China).

2. ^{1}H NMR analysis for *β*-CD decoration

^{1}H NMR spectrum of *β*-CD and its derivative *β*-CD-MA were recorded on a Bruker Avance III 500M Hz spectrometer in DMSO-d6 at 25 °C. Majority of the spectroscopy of *β*-CD-MA was consistent with *β*-CD, while the appearance of peaks at 5.97 ppm (s, -OOCC(CH_3)C$\boldsymbol{H_2}$-, a_1), 5.60 ppm (s, -OOCC(CH_3)C$\boldsymbol{H_2}$-, a_2), and 1.84 ppm (s, -OOCC(C$\boldsymbol{H_3}$)CH_2-, b) indicated the successful introduction of vinyl groups. However, the ratio of peak area between the characterized 1-H (δ = 4.83 ppm) and a_1/a_2-H (δ = 5.97 or 5.60 ppm) was about 21, demonstrated that only one acrylic group was grafted on per three *β*-CD. The derivatization took place on 6-OH rather than 2-OH, or 3-OH, which led to its slight declination (δ = 4.44), as well as slight chemical shift increase on part of 6-H, form 3.62 ppm to 4.83 ppm (**Figure S2**).

3. HPLC-MS/MS Analysis

An Agilent 1260 HPLC system (Agilent Technologies, CA, USA) coupled to an Triple Quad 4500 triple-quadrupole tandem mass spectrometer with an ESI source (Applied Biosystems/MDS Sciex, MA, USA) was operated in the negative ion mode. A Zorbax SB-C18 column (2.1 mm × 150 mm, 3.6 μm, Agilent Technologies, CA, USA) was used for separation, with 0.01% formic acid in water (solvent A) and 0.01% formic acid in acetonitrile (solvent B) as the mobile phases. The flow rate was set at 400 μL·min-1, and the column was maintained at 45 ℃.The optimized gradient and the monitoring transition of BAs were listed in Table S1, and the detail transitions of eight BAs can be found in Table S2.

4. Verification of the Sampling-rate Calibration Method

Theoretically, at a certain duration before reaching the equilibrium for SPME, R_s is a function of the physicochemical properties of the sample matrix, extraction phase, target analytes as well as the matrix temperature, but independent from the concentration of analytes (C_s) and extraction time (t).[1] Therefore, sampling-rates of eight BAs from buffer solution to the copolymer probe were obtained by dividing the slope of n-C_s curves (**Figure S4**) by t. The derived R_s values were listed in Table 2. Then, extractions were operated in 20 ng ml^{-1} mixing solutions for 10 min and 50 min. The results showed high consistency with those values obtained according to Equation 2 (**Figure S5**), indicating the feasibility of the present method.

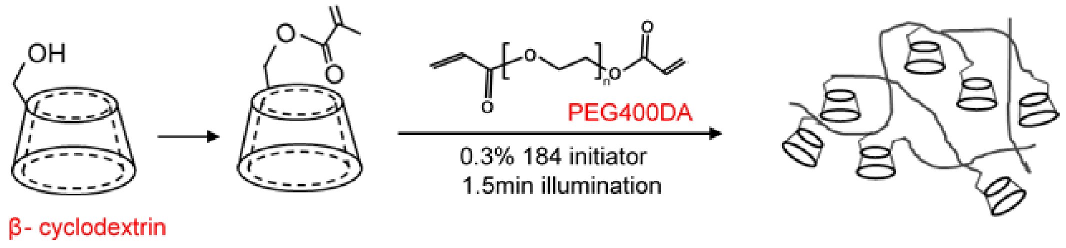

Figure S1. Scheme of the derivatization of β-CD and the following polymerization.

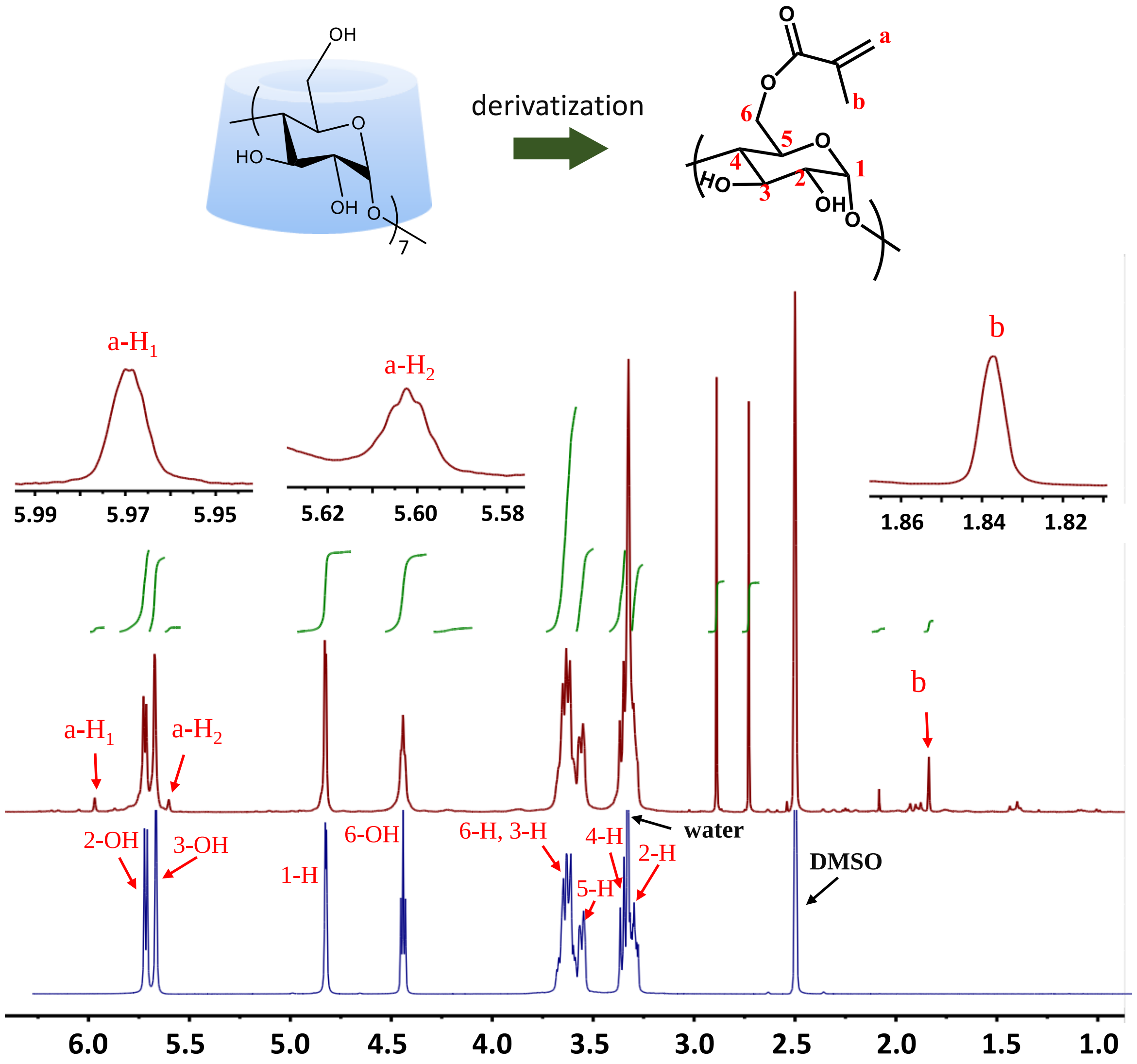

Figure S2. ^{1}H NMR spectrum of *β*-CD (blue line) and *β*-CD-MA (red line) at 25 °C. Scheme at top illustrated the possible derivation position on *β*-CD. For *β*-CD-MA, ^{1}H NMR (500 MHz, DMSO-d_6, δ): 5.97 (s, 1H, a-H_1), 5.73 (t, J = 8.9 Hz, 21H, 2-OH), 5.67 (s, 21H, 3-OH), 5.60 (s, 1H, a-H_2), 4.83 (d, J = 3.2 Hz, 22H, 1-H), 4.44 (t, J = 5.0 Hz, 20H, 6-OH), 3.62 (dd, J = 17.0, 7.9 Hz, 62H, 6-H and 3-H), 3.56 (d, J = 9.3 Hz, 21H, 5-H), 3.36 (d, J = 9.3 Hz, 21H, 4-H), 3.29 (d, J = 9.7 Hz, 18H, 2-H), 1.84 (s, 3H, b-H); for β-CD, ^{1}H NMR (500 MHz, DMSO-d_6, δ): 5.72 (d, J = 6.9 Hz, 1H, 2-OH), 5.67 (d, J = 2.0 Hz, 1H, 3-OH), 4.83 (d, J = 3.4 Hz, 1H, 1-H), 4.44 (t, J = 5.6 Hz, 1H, 6-OH), 3.74 – 3.58 (m, 3H, 6-H and 3-H), 3.56 (d, J = 9.7 Hz, 1H, 5-H), 3.36 (d, J = 9.3 Hz, 1H, 4-H), 3.32 – 3.27 (m, 1H, 2-H).

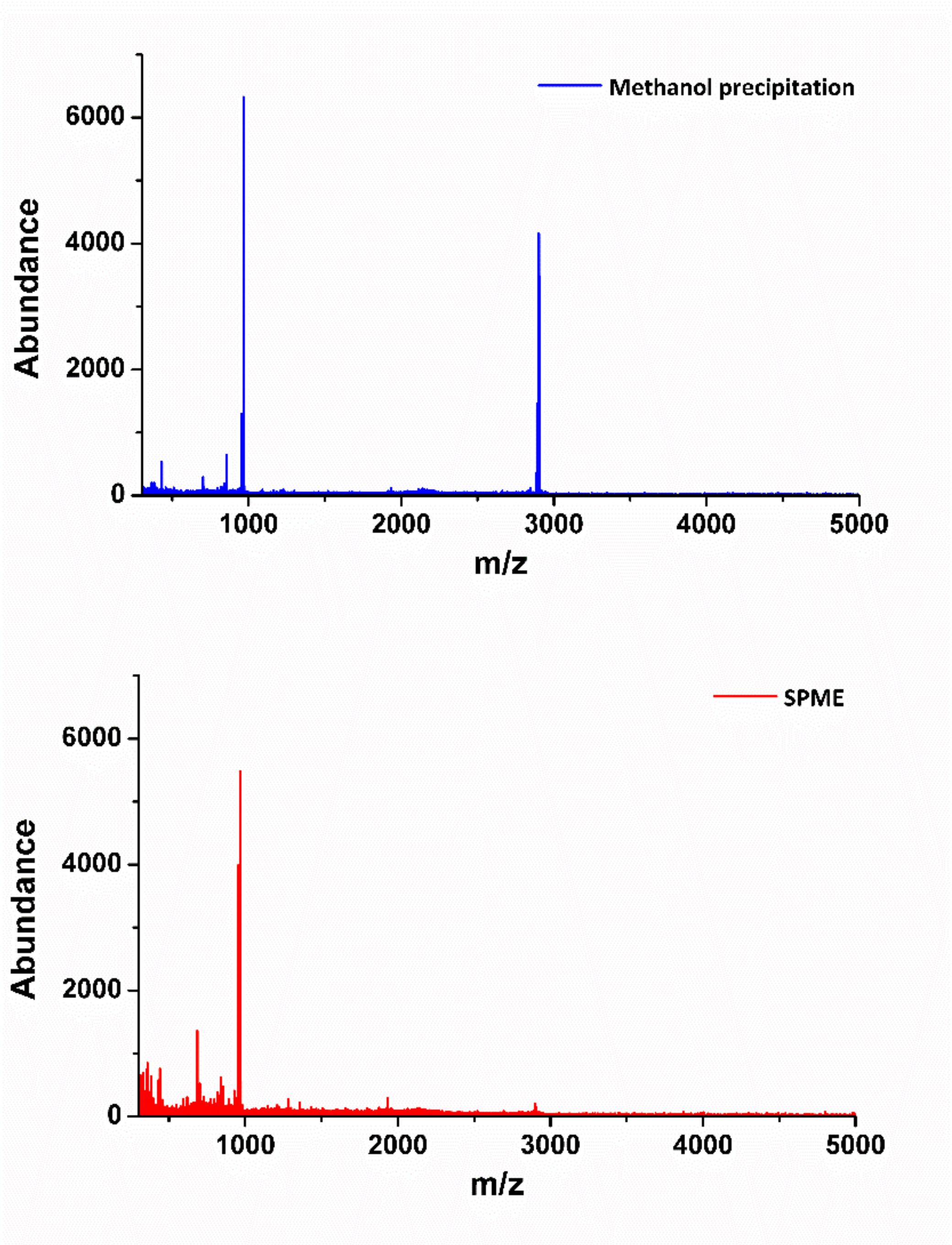

Figure S3. MALDI-TOF analysis (m/z from 300-5000) of human urine sample after traditional methanol precipitation and the SPME method developed in the present work.

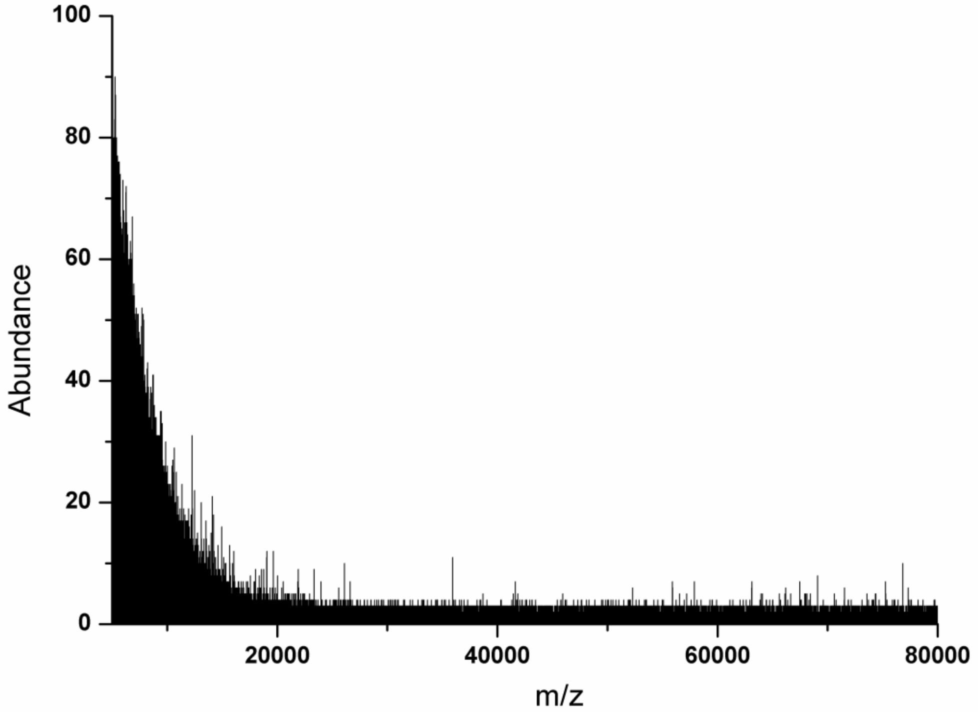

Figure S4. The biological macromolecule analysis of the eluent using MALDI-TOF-MS (m/z from 5000-80000).

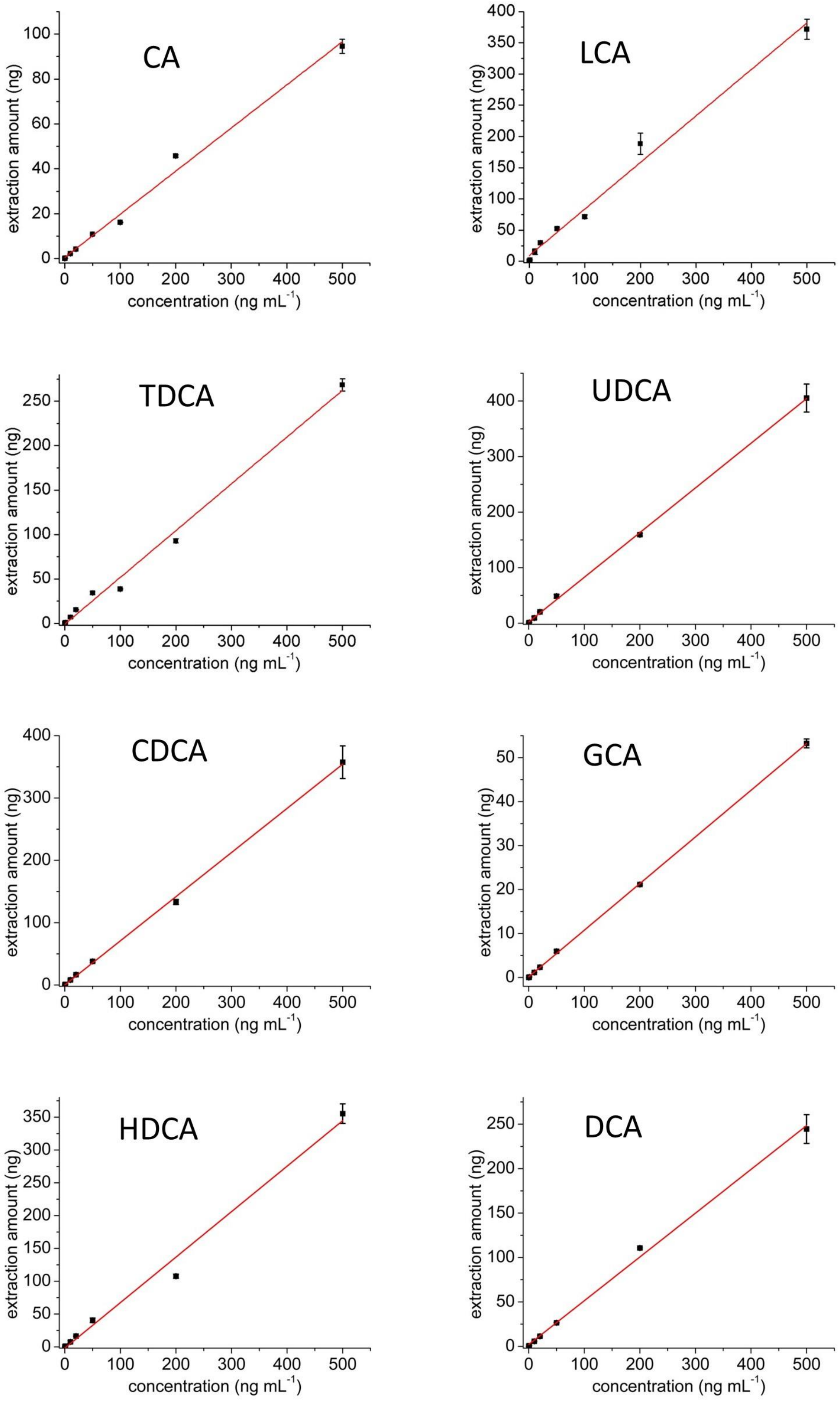

Figure S5. The standard lines (*n*-C_s curves) of SPME in BAs solutions (*t*=30 min, n=6).

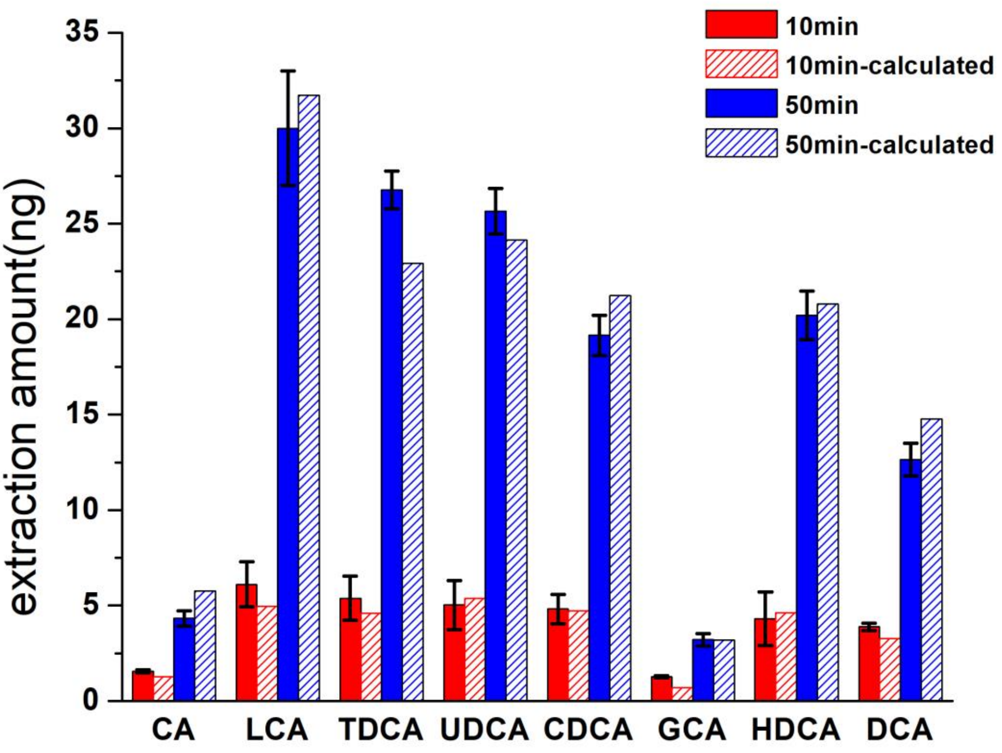

Figure S6. The extraction amounts obtained by experiments and calculations according to the Sampling-rates. (n=6 for experimental data).

Table S1. The comparison of limits of detection (LODs) between the present method and others in reported works.[a)]

Ref.	Analyzation technique	Sample preparation method	unit	CA	GCA	UDCA	CDCA	HDCA	DCA	TUDCA	LCA
2	GC-MS/MS	SPE	ng mL^{-1}	0.80	-	0.80	0.60	1.60	0.40	-	0.40
3	LC-MS/MS	SPME	nmol L^{-1}	0.007	-	-	-	-	-	-	-
4[b)]	LC-MS/MS	SPE	μM	0.180	0.060	0.180	0.360	0.300	0.360	0.060	-
5	GC-MS	SPE	ng g^{-1}	-	-	3.0	2.8	4.0	1.8	-	0.5
6	LC/MS-IT-TOF	HF-LPME	ng mL^{-1}	1.00	-	1.00	-	1.00	1.00	-	1.00
7	UHPLC-MS/MS	LE	pg mL^{-1}	35	10	150	140	200	110	40	350
8	LC-MS/MS	LE	nmol mL^{-1}	0.012	0.104	0.017	0.012	0.016	0.019	0.038	0.017
9	UHPLC-MS/MS	LE	nM	0.5	5	0.5	1	0.25	0.25	5	0.25
**this work**	**HPLC-MS/MS**	**SPME**	**ng mL^{-1}**	**0.028**	**0.056**	**0.036**	**0.075**	**0.026**	**0.028**	**0.003**	**0.015**

[a)] "-" in the table refers not mentioned in the reference; [b)] Statistic listed in the table refers to the limits of quantification (LOQs) of the method.

Table S2. The concentration of eight target BAs in human urine sample and their recoveries. (n=6)

Analytes	C (ng mL^{-1}) [a)]	Recoveries (%)
CA	48.93±4.79	108.47
LCA	ND	93.42
TUDCA	1.53±0.55	104.94
UDCA	ND	80.74
CDCA	0.88±0.37	90.54
GCA	32.51±2.74	102.02
HDCA	2.71±1.57	87.05
DCA	3.31±0.93	98.58

[a)] ND means the detected concentration was lower than the limit of quantitation.

Table S3. The mobile phases gradients for HPLC-MS/MS analysis. Solvent A refers to water with 0.01% formic acid and Solvent B is acetonitrile with 0.01% formic acid.

Time (min)	Solvent A (%)	Solvent B (%)
0	66	34
15	60	40
18	57	43
19	50	50
27	50	50
28	0	100
32	0	100
33	66	34
40	66	34

Table S4. The MRM transitions and corresponding ionized parameters of eight BAs used in this work.

Analytes	Q1 Mass (m/z)	Q3 Mass (m/z)	DP (V)	EP (V)	CE (V)	CXP (V)
**Unconjugated bile acids**						
CA	407.3	343.10	-130	-11	-43	-11
DCA	391.3	391.30	-125	-10	-15	-8
HDCA	391.3	391.20	-125	-10	-15	-8
UDCA	391.3	391.20	-125	-10	-15	-8
CDCA	391.3	391.20	-125	-10	-10.5	-8
LCA	375.2	375.20	-95	-10	-15	-14
**Conjugated bile acids**						
GCA	464.2	73.80	-160	-10	-38	-13
TUDCA	498.2	79.70	-150	-10	-135	-12

References

[1] J. Ai, *Anal. Chem.* **1997**, *69*, 1230.

[2] S. J. J. Tsai, Y. S. Zhong, J. F. Weng, H. H. Huang, P. Y. Hsieh, *J. Chromatogr. A* **2011**, *1218*, 524.

[3] V. Bessonneau, B. Bojko, A. Azad, S. Keshavjee, S. Azad, J. Pawliszyn, *J. Chromatogr. A* **2014**, *1367*, 33.

[4] C. John, P. Werner, A. Worthmann, K. Wegner, K. Tödter, L. Scheja, S. Rohn, J. Heeren, M. Fischer, *J. Chromatogr. A* **2014**, *1371*, 184.

[5] J. J. Birk, M. Dippold, G. L. Wiesenberg, B. Glaser, *J. Chromatogr. A* **2012**, *1242*, 1.

[6] M. J. N. de Paiva, H. C. Menezes, J. C. C. da Silva, R. R. Resende, Z. de Lourdes Cardeal, *J. Chromatogr. A* **2015**, *1388*, 102.

[7] S. E. Jäntti, M. Kivilompolo, L. Öhrnberg, K. H. Pietiläinen, H. Nygren, M. Orešič, T. Hyötyläinen, *Anal. Bioanal. Chem.* **2014**, *406*, 7799.

[8] X. Qiao, M. Ye, D. Pan, W. Miao, C. Xiang, J. Han, D. Guo, *J. Chromatogr. A* **2011**, *1218*, 107.

[9] M. H. Sarafian, M. R. Lewis, A. Pechlivanis, S. Ralphs, M. J. W. McPhail, V. C. Patel, M. Dumas, E. Holmes, J. K. Nicholson, *Anal. Chem.* **2015**, *87*, 9662.
